# Successful reconstruction using a buccal fat pad flap in misdiagnosed buccinators intramuscular synovial sarcoma: A case report

**DOI:** 10.1097/MD.0000000000035966

**Published:** 2023-11-10

**Authors:** Da Woon Lee, Hyun Kim, Jang Si-Hyong, Je Yeon Byeon, Hwan Jun Choi

**Affiliations:** a Department of Plastic and Reconstructive Surgery, College of Medicine, Soonchunhyang University Cheonan Hospital, Cheonan, Korea; b Department of Pathology, College of Medicine, Soonchunhyang University Cheonan Hospital, Cheonan, Korea.

**Keywords:** buccal fat pad, case report, management, oral cavity, synovial sarcoma

## Abstract

**Introduction:**

Synovial sarcoma (SS) is a subtype of soft tissue sarcoma that primarily usually occurs in the lower extremities but rarely arises in the head and neck areas, including the oral cavity. Due to its variable presentation and similarity to benign masses in terms of age at onset, growth rate, and favorable outcomes, SS is often misdiagnosed as a benign tumor. However, it is a malignant tumor.

**Patient concerns:**

We report the case of intramuscular SS in the oral cavity. Initially, the lesion was clinically suspected as a benign mass but was ultimately confirmed as malignant SS.

**Diagnosis:**

Although histopathological examination is the first step in diagnosing SS, molecular testing to confirm the presence of SYT-SSX fusion can provide a definitive diagnosis when the histopathology is inconclusive. In this patient as well, the postoperative pathological report confirmed the diagnosis of biphasic SS, and molecular testing revealed positive SYT/SSX fusion.

**Therapeutics interventions:**

Following the recommendation of multidisciplinary care system, a wide excision was performed including the buccinators muscle, and reconstruction was performed using a buccal fat pad flap to prevent cheek depression.

**Outcomes:**

On the final pathologic report, SS was removed margin-free, and there were no metastatic lymph nodes. No evidence of cheek dimpling was observed, and follow-up neck CT showed no significant changes in the lymph nodes. As a result of observation up to several months after surgery, there were no functional and aesthetic complications.

**Conclusions:**

We report a successful case of intramuscular SS resection, initially misdiagnosed as a benign mass, using a buccal fat pad flap. We also highlight the importance of correctly diagnosing SS, especially in the craniofacial region where it can be mistaken for benign masses.

## 1. Introduction

Synovial sarcoma (SS) accounts for 5.6% to 10% of all soft tissue sarcomas (STS) and primarily arises in the large joint capsules and articular tendons of the lower limbs.^[1]^ In the United States, it predominantly affects individuals between the ages of 15 and 40 years, with an estimated incidence of 0.81/1000,000 in children and 1.42/1000,000 in adults.^[2]^ Among pediatric STS, SS is one of the most common non-rhabdomyosarcomas, comprising 30% of cases.^[2]^ Although SS commonly arises in the joints, it can also occur at sites unrelated to synovial tissue, leading to uncertainty regarding its exact origin. The SS exhibits variable morphological and genetic characteristics. It often occurs in young patients, exhibits slow growth over several months after the initial presentation, lacks an invasive pattern, and primarily forms in deep tissues, resulting in frequent confusion with benign tumors. The average duration of SS before diagnosis is approximately 2 years.^[3]^ Unlike other STS, SS has a longer duration of persistent symptoms, including prolonged joint pain and dysfunction.^[4]^ Among SS cases, only 3% to 10% occur in the head and neck area and are primarily confined to the parapharyngeal space.^[5]^ Intramuscular SS in the oral cavity is extremely rare, with only a few dozen cases reported to date.^[6]^ For these reasons, SS in the oral cavity is prone to misdiagnosis and often remains undiagnosed until the disease has progressed significantly.

The buccal fat pad (BFP) serves multiple functional roles, such as acting as a cushion between the fascial space and facial muscles, assisting in muscle movement, separating mastication, and maintaining the negative pressure required for suction in newborns.^[7]^ The BFP flap has been widely employed in defect reconstruction not only in the oral cavity but also in the maxilla and nasal cavity. The BFP flap offers the advantages of low morbidity, fewer complications, and ease of surgical technique.^[8]^

Here, we report the case of a male patient diagnosed with intramuscular SS in the oral cavity. Initially, the lesion was provisionally diagnosed as a benign mass resembling a fibroma but was ultimately confirmed as SS. Considering its rarity, this case makes a valuable contribution to the literature and provides an opportunity for comparison with previous cases reported at the same location, thereby contributing to the exploration of optimal treatment options. Furthermore, the treatment for this condition requires a multidisciplinary approach, underscoring the importance of such an approach in determining management strategies. A multidisciplinary approach is important when considering the best treatment options for rare conditions, such as intramuscular SS in the oral cavity.

## 2. Patients and methods

This retrospective case study involved a patient who visited our outpatient clinic (Department of Plastic and Reconstructive Surgery). The study protocol was approved by our Institutional Review Board (number: 2023-14-002). The patient provided informed consent for the publication of the case. All procedures were performed in accordance with the ethical standards of the institutional and/or national research committee and the 1964 Declaration of Helsinki and its later amendments or comparable ethical standards. Considering that the patient was a child, oral and written consent was obtained from the patient’s mother. As for the Data Availability Statement, data sharing is not applicable to this article as no datasets were generated or analyzed during the current study. Because this article is a case report.

## 3. Case report

**Figure 1.**
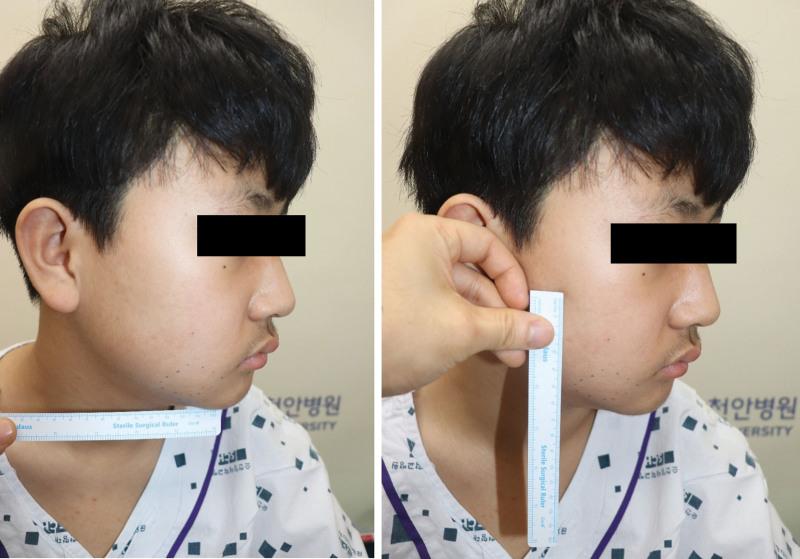
Preoperative findings. A protruding mass on the cheek is observed, indicated by a dotted line.

An 11-year-old boy presented to our outpatient department with a right cheek mass that had appeared 4 months prior. On presentation, the mass was characterized as hard and non-tender (Fig. 1). The patient reported no other symptoms related to the mass. Intraoral examination revealed a 1 × 2 × 1 cm-sized mass.

**Figure 2.**
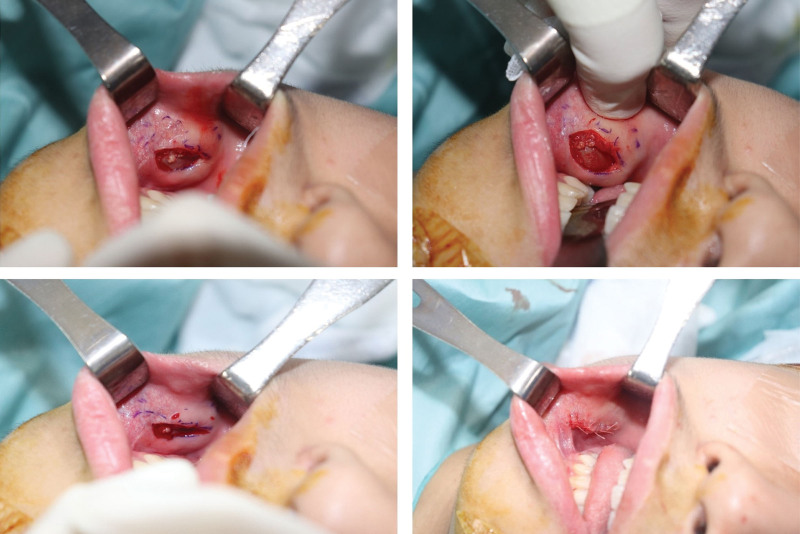
Intraoperative findings during the first operation (excisional biopsy). Exposure of the buccinators muscle.

Initially, considering the possibility of an intraoral fibroma or mucoid cyst, a simple excisional biopsy was performed. Intraoperatively, the mass was oval-shaped, located within the buccinator muscle, and attached to the muscle fascia. No capsule formation was observed, and there were no connections to major structures such as the buccal branch or Stensen duct (Fig. 2). The postoperative pathological report confirmed the diagnosis of biphasic SS, and molecular testing revealed positive SYT/SSX fusion.

**Figure 3.**
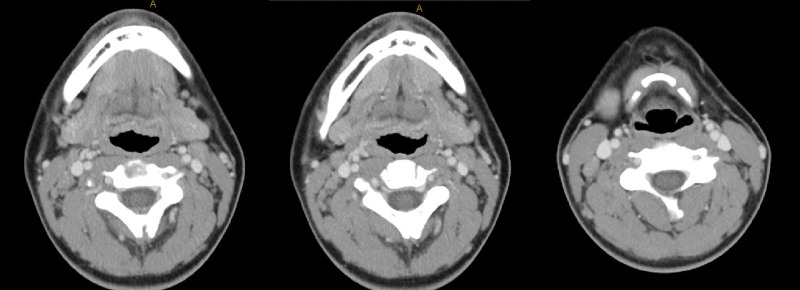
Neck CT. Reactive lymph nodes are observed in the neck level II–III, but no pathological findings are present. CT = computed tomography.

**Figure 4.**
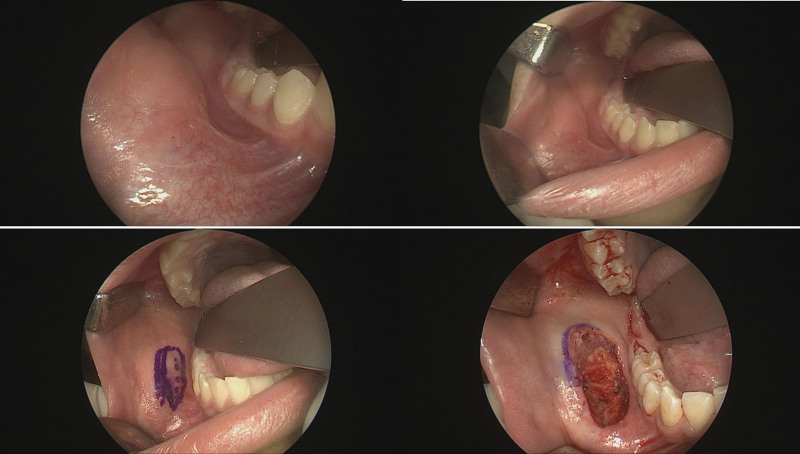
Intraoperative findings during the second operation (wide excision). A 5 mm free margin was achieved.

**Figure 5.**
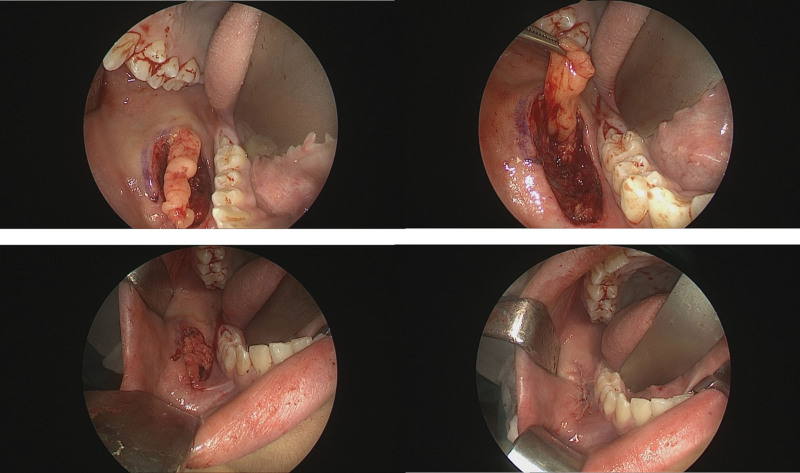
Appearance after the second operation, utilizing a pedicled buccal fat pad flap to cover the depressed defect and prevent dimpling.

Positron emission tomography/computed tomography (PET/CT) was performed to assess regional or distant metastases. Definite fluorodeoxyglucose uptake was not observed in the right oral cavity or cheek, but reactive lesions were found in both neck level II lymph nodes. These lymph nodes appeared reactive rather than pathological on neck CT (Fig. 3). According to the 2022 National Comprehensive Cancer Network soft tissue sarcoma guidelines, the patient was classified as having stage 1A T1bN0M0. Wide excision confirming free margins was recommended by a multidisciplinary care team that included clinicians from the plastic surgery, pathology, and oncology departments. Given the patient’s pediatric status and the absence of a defined treatment protocol for SS, a wide excision was performed with a 5 mm free margin (Fig. 4). A biopsy was also performed on one of the reactive lymph nodes observed on PET/CT and neck CT. Pathological frozen biopsy confirmed that all surgical margins were negative. Following a wide excision, significant dimpling occurred in the oral cavity due to scarring and fat atrophy, necessitating coverage with a BFP flap (Fig. 5). A local advancement flap with bilateral flap elevation was placed in the oral mucosa to cover the defect site.

**Figure 6.**
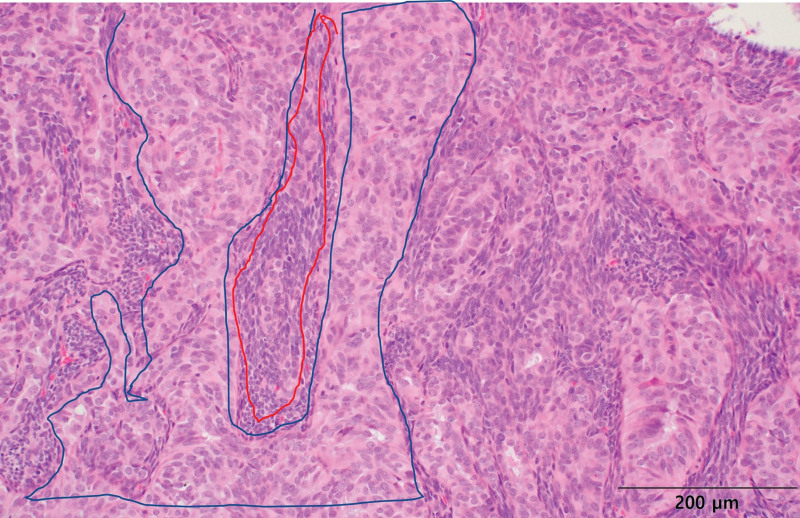
Pathologic findings. The tumor exhibited biphasic histological features consisting of spindle and epithelial cells (hematoxylin and eosin stain).

**Figure 7.**
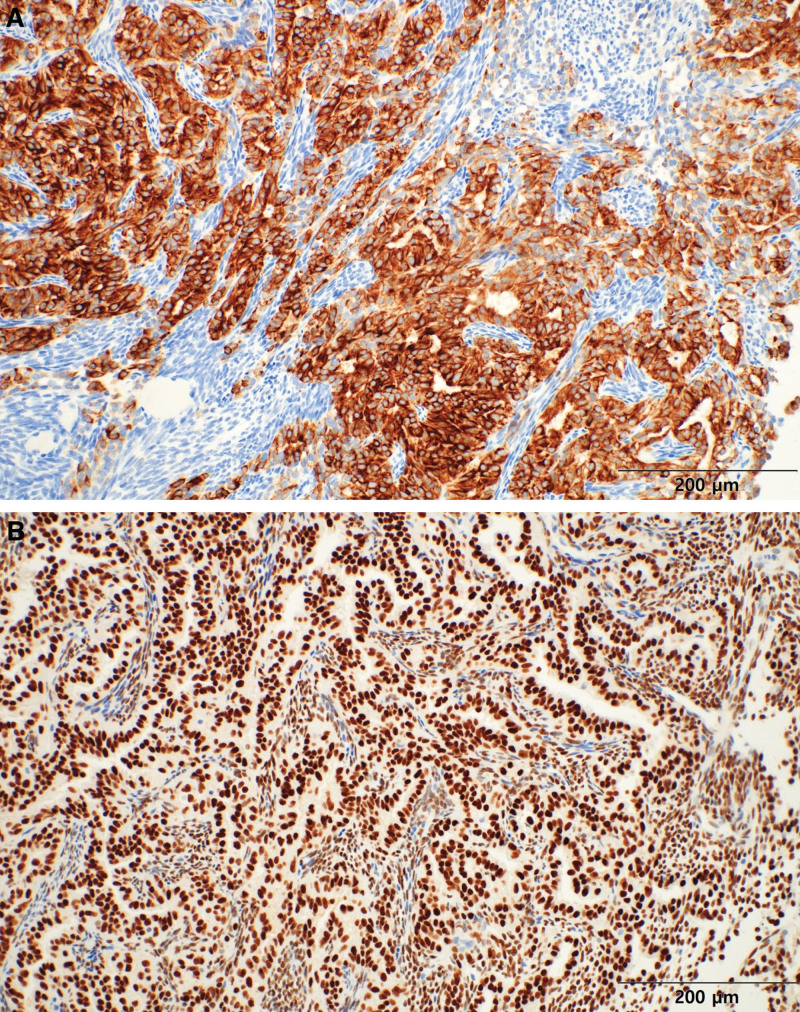
Immunohistochemistry staining findings. (A) The epithelial component was positive for pan-CK, while the spindle cell component was negative. (B) TLE1 immunohistochemistry demonstrated diffuse strong positivity in all tumor cells.

After a wide excision, the pathological report confirmed the diagnosis of SS, which was consistent with previous findings. Furthermore, it was confirmed that the additional cervical lymph nodes were not metastatic. According to the pathological report, microscopic examination of the excised tumor revealed a solid growth pattern with increased cellularity. The tumor exhibited biphasic morphology consisting of spindle cells and gland-like epithelial cells. The epithelial cells displayed moderate eosinophilic cytoplasm and round to ovoid nuclei with occasional small conspicuous nucleoli, whereas the spindle cells exhibited evenly distributed chromatin with inconspicuous nucleoli (Fig. 6). Immunohistochemical staining for pan-CK was positive for epithelial cells but negative for spindle cells (Fig. 7A). Strong diffuse positivity for both the epithelial and spindle cell components was observed by immunohistochemical staining for TLE1 (Fig. 7B). Furthermore, the presence of SYT-SSX gene fusion transcripts was confirmed by reverse transcription polymerase chain reaction, providing evidence for the diagnosis of SS.

**Figure 8.**
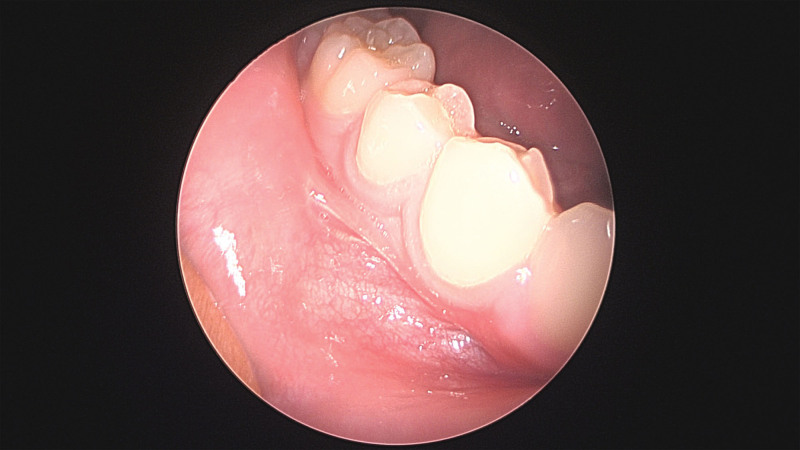
Photographic findings one month after surgery. No dimpling or wound problems were observed in the cheek or oral mucosa.

On postoperative day one, there were no functional problems such as trismus or wound complications (Fig. 8). No evidence of dimpling was observed in the external oral cheek, and follow-up CT of the neck showed no significant changes in the lymph nodes.

## 4. Discussion

The BFP is an encapsulated adipose tissue mass located between the buccinator muscle, mandibular ramus, and masseter muscle. It receives a rich blood supply from the maxillary, superficial temporal, and facial arteries, making it suitable for molding to match the defect shape.^[9]^ The BFP has 4 extensions: the central body, buccal, pterygoid, pterygopalatine, and temporal extensions, with the central body and buccal extension accounting for approximately 50% of the total volume.^[10–12]^ In general, the central body and buccal extension are components of the BFP flap and are transposed to cover the oral cavity defects. The average volume of the BFP was 10 mL, with an average weight of 10 g.^[13]^ Tideman et al^[14]^ reported that a BFP can cover defects of up to 6 × 5 × 3 cm, but the typical dimensions for coverage are considered to be approximately 4 × 4 × 3 cm.^[15]^ This is because the flap should be sutured to the defect margin without tension to prevent necrosis.

The BFP flap is easy to harvest, provides reliable surgical results, and is associated with few major complications.^[16]^ Possible complications of pedicled BFP flaps include the development of a cheek dimple when a large amount of BFP is harvested^[10]^ and unexpected trismus at the flap donor site.^[17]^ A BFP flap for palatal and alveolar defect reconstruction can also result in flap perforation, which can be minimized by maintaining a liquid or soft diet for 4 to 6 weeks until the BFP epithelizes.^[9]^

SS was first described by Simon in 1865 and clinically named by Sabrazes et al in 1934.^[18]^ SS is a tumor with a relatively slow growth rate and favorable prognosis. Although the diagnosis of SS is not unique, magnetic resonance imaging can be performed to determine the primary site, the extent of adjacent tissue invasion, and lymph node metastasis. Gadolinium contrast can help differentiate between areas of hemorrhage or necrosis surrounding a solid tumor mass.^[19]^ Similar to most STS, SS appears low-intensity on T1-weighted images and high-intensity with gadolinium enhancement on T2-weighted images.^[20]^

SS do not have specific histological markers. Therefore, several antibody markers have been used for diagnosis. Histologically, SS can be classified into 3 types, with monophasic fibrous or monophasic epithelial being the most common (approximately 70%), followed by biphasic (25%), with the remaining cases exhibiting poorly differentiated or myxoid patterns.^[21]^ In this case, biphasic SS demonstrates 2 separate populations of epithelial-like cells in the glandular structures and spindle cells.^[22]^ However, biphasic morphology alone is insufficient to diagnose biphasic SS.

Immunohistochemical staining for specific markers varies in cases with SS. However, chromosomal studies have consistently shown a balanced reciprocal translocation t(X;18) (p11.2q;q11.2). This translocation leads to the fusion of SYT or SSXT on chromosome 18 with SSX1, SSX2, or SSX4 on the X chromosome. Therefore, SYT-SSX fusion plays a crucial role in the diagnosis of SS.^[23]^ In the present case, although SS could not be diagnosed based on histopathological findings alone, the positive SYT-SSX fusion confirmed the diagnosis of SS.

There is no established treatment protocol for SS, including head and neck SS. However, surgical resection, including wide excision of the primary lesion to secure a free margin, is essential. In cases where complete surgical resection with a free margin is not feasible, or when necessary, adjuvant radiation therapy may be considered. Adjuvant chemotherapy is also recommended, although its indications are limited.^[24]^ According to recent debates, lymphatic spread is uncommon in SS; therefore, prophylactic cervical lymph node dissection is not recommended.^[25,26]^

Appropriate treatment of SS and other STS requires a multidisciplinary approach. Decisions regarding staging, pathological evaluation, and adjuvant therapy should be made in close collaboration among various fields. In particular, head and neck STS often present challenges in achieving complete resection. Complete resection is difficult when the tumor grows functionally over multiple areas. Generally, SS is known for its high recurrence rate owing to the lack of clear encapsulation and abundant blood supply in the head and neck area.^[27]^ In the present case, the tumor was detected relatively early, allowing for complete removal without functional deficits. However, oral cavity tumors, especially intramuscular tumors, are often difficult to diagnose, and complete resection may not be feasible.^[28]^ Although this patient had a low stage and small tumor size, indicating a favorable prognosis, SS generally has a poor prognosis in older individuals or cases with poor differentiation and tumor size > 5 cm.^[29]^ Notably, the 5-year survival rate of SS in children was significantly lower than that in adults.^[2,30,31]^

In this case report, there are 2 limitations. First, only one case was presented, so various aspects of SS could not be demonstrated. This may also be a bias that oral SS can show a favorable course. However, progressed SS is often reported to be unresectable, and the prognosis is poor.^[28]^ Second, follow-up results after surgery have not been presented for a long time, so it is impossible to completely exclude the possibility of recurrence. Photographic and PET/CT images of the lesion are required several months after operation.

## 5. Conclusion

SS is known for its deceptive clinical presentation, often mimicking benign masses that can lead to misdiagnosis and inappropriate management. In the craniofacial region, where functional and aesthetic considerations are critical, an accurate diagnosis is of utmost importance.

This report presents a rare and valuable case within the realm of rare sarcomas, demonstrating unique pathological findings and emphasizing the importance of accurate diagnosis in the management of oral mucosal tumors. This case holds significance due to its rarity and can provide insights into the pathological diagnosis of SS. Due to the challenging nature of early detection and the potential for widespread metastasis of oral mucosal tumors, it is crucial to differentiate such tumors accurately and make a proper diagnosis. The BFP flap proved to be a useful technique for achieving convenient coverage of the buccinator and cheek defects.

## Author contributions

**Conceptualization:** Da Woon Lee, Hyun Kim, Je Yeon Byeon, Hwan Jun Choi.

**Data curation:** Jang Si-Hyong, Je Yeon Byeon, Hwan Jun Choi.

**Formal analysis:** Jang Si-Hyong.

**Project administration:** Hwan Jun Choi.

**Supervision:** Da Woon Lee, Hwan Jun Choi.

**Visualization:** Jang Si-Hyong.

**Writing – original draft:** Da Woon Lee, Hyun Kim.

**Writing – review & editing:** Da Woon Lee, Hyun Kim, Je Yeon Byeon.
